# DNA methylation in a Scottish family multiply affected by bipolar disorder and major depressive disorder

**DOI:** 10.1186/s13148-016-0171-z

**Published:** 2016-01-20

**Authors:** Rosie May Walker, Andrea Nikie Christoforou, Daniel L. McCartney, Stewart W. Morris, Nicholas A. Kennedy, Peter Morten, Susan Maguire Anderson, Helen Scott Torrance, Alix Macdonald, Jessika Elizabeth Sussmann, Heather Clare Whalley, Douglas H. R. Blackwood, Andrew Mark McIntosh, David John Porteous, Kathryn Louise Evans

**Affiliations:** Medical Genetics Section, Centre for Genomic and Experimental Medicine, Institute of Genetics and Molecular Medicine, The University of Edinburgh, Western General Hospital, Crewe Road, Edinburgh, EH4 2XU UK; Division of Psychiatry, The University of Edinburgh, Royal Edinburgh Hospital, Edinburgh, UK; Centre for Cognitive Ageing and Cognitive Epidemiology, The University of Edinburgh, 7 George Square, Edinburgh, EH8 9JZ UK

**Keywords:** Bipolar disorder, Major depressive disorder, DNA methylation, 450K array, FANCI, Gene ontology analysis, Family study

## Abstract

**Background:**

Bipolar disorder (BD) is a severe, familial psychiatric condition. Progress in understanding the aetiology of BD has been hampered by substantial phenotypic and genetic heterogeneity. We sought to mitigate these confounders by studying a multi-generational family multiply affected by BD and major depressive disorder (MDD), who carry an illness-linked haplotype on chromosome 4p. Within a family, aetiological heterogeneity is likely to be reduced, thus conferring greater power to detect illness-related changes. As accumulating evidence suggests that altered DNA methylation confers risk for BD and MDD, we compared genome-wide methylation between (i) affected carriers of the linked haplotype (ALH) and married-in controls (MIs), (ii) well unaffected haplotype carriers (ULH) and MI, (iii) ALH and ULH and (iv) all haplotype carriers (LH) and MI.

**Results:**

Nominally significant differences in DNA methylation were observed in all comparisons, with differences withstanding correction for multiple testing when the ALH or LH group was compared to the MIs. In both comparisons, we observed increased methylation at a locus in *FANCI*, which was accompanied by increased FANCI expression in the ALH group. *FANCI* is part of the Fanconi anaemia complementation (*FANC*) gene family, which are mutated in Fanconi anaemia and participate in DNA repair. Interestingly, several *FANC* genes have been implicated in psychiatric disorders. Regional analyses of methylation differences identified loci implicated in psychiatric illness by genome-wide association studies, including *CACNB2* and the major histocompatibility complex. Gene ontology analysis revealed enrichment for methylation differences in neurologically relevant genes.

**Conclusions:**

Our results highlight altered DNA methylation as a potential mechanism by which the linked haplotype might confer risk for mood disorders. Differences in the phenotypic outcome of haplotype carriers might, in part, arise from additional changes in DNA methylation that converge on neurologically important pathways. Further work is required to investigate the underlying mechanisms and functional consequences of the observed differences in methylation.

**Electronic supplementary material:**

The online version of this article (doi:10.1186/s13148-016-0171-z) contains supplementary material, which is available to authorized users.

## Background

Bipolar disorder (BD) is severe psychiatric condition characterised by recurrent episodes of depression and mania. BD is highly heritable (*h*^2^ = 0.85 [1]), and in recent years, progress has been made in identifying risk-associated variants through genome-wide association studies (GWASs) (reviewed in [2]). These risk variants are, however, of small effect and together only partially explain BD’s heritability [3, 4].

Many of the associated variants for BD fall within non-coding regions of the genome [5], implicating perturbed regulatory processes in the pathogenesis of this condition. Consistent with this observation, gene expression studies have identified several changes in individuals with BD [6, 7].

Some of these expression changes might be attributable to altered DNA methylation, which has been observed in individuals with BD, for example [8–12]. DNA methylation levels at many sites are, at least in part, under genetic control [13], indicating one potential mechanism by which regulatory variants might confer risk for BD. In addition, comparison of monozygotic twins who are discordant for BD, or the related psychiatric illness schizophrenia, has revealed several loci showing significant differences in DNA methylation [14]. These differences might reflect the actions of environmental risk factors, which are believed to contribute to the imperfect concordance for BD observed in monozygotic twins.

To date, case-control studies of DNA methylation in BD have been limited either by a restricted focus on candidate genes [8–10, 12] or the study of a small group of unrelated individuals [15, 16]. Here, we capitalise on the increase in homogeneity conferred by the study of related individuals by assessing blood-based DNA methylation in a large Scottish family multiply affected by BD and major depressive disorder (MDD). Increased aetiological homogeneity within this family may confer greater statistical power to detect phenotypically relevant differences in DNA methylation. Previous analyses of this family have identified a ~20 Mb haplotype on chromosome 4p15-16, which shows linkage to BD and MDD (henceforth referred to as the linked haplotype (LH)) [17, 18] (maximum LOD score = 4.41). Ongoing analysis of the family, which now includes additional individuals, suggests that the LH acts in conjunction with genome-wide polygenic risk to confer risk for BD (Clarke et al., manuscript in preparation).

Here, we assessed the hypothesis that the LH confers risk via an effect on DNA methylation and identified four loci showing differences in methylation in carriers of the LH that withstood correction for multiple testing. Furthermore, as some carriers of the LH do not develop BD or MDD, we attempted to identify differences in DNA methylation that represent additional risk and/or protective loci that act in concert with the linked haplotype to determine an individual’s phenotype. In this comparison, no individual locus remained significant after correction for multiple testing; however, collectively, the most differentially methylated loci were found to map to genes involved in neurologically relevant functions. Together, these analyses aimed to assess the contribution of genetically and environmentally driven changes in DNA methylation to the development of BD and MDD.

## Results

### Overview of research strategy

Genome-wide DNA methylation was measured in members of a large family multiply affected by BD and MDD, using the Infinium HumanMethylation450 BeadChip. Three groups of individuals were considered: affected (diagnosed with either BD or MDD) carriers of the linked haplotype (ALH; *n* = 10; (BD *n* = 5; MDD *n* = 5)); unaffected carriers of the linked haplotype (ULH; *n* = 10) and unaffected, non-haplotype-carrying married-in controls (MIs; *n* = 9). A series of comparisons was performed: ALH vs. MI, ULH vs. MI, ALH vs. ULH and LH (combined ALH and ULH) vs. MI. These comparisons permitted us to assess our hypotheses that (i) the linked haplotype confers an increase in risk for major affective disorders via an effect on DNA methylation and (ii) DNA methylation at certain loci correlates with the presence/absence of illness in haplotype carriers, reflecting the involvement of additional risk/protective loci. To gain an insight into the potential consequences of differential methylation, gene ontology analysis was carried out to identify biological processes and functions overrepresented amongst the most significantly differentially methylated loci.

### Quality control and data filtering

As the performance of some probes on the array is known to be affected by the presence of single nucleotide polymorphisms (SNPs) or cross-hybridisation, these probes were removed prior to data analysis. Firstly, 17,955 probes in which the target CpG is located within 2 bp of a SNP (minor allele frequency ≥5 %) and 29,093 probes predicted to cross-hybridise [19] were removed from the dataset. The overall success of DNA methylation profiling was then assessed by considering plots representing the output of quality control probes, which measure the success of various stages of the profiling process. This revealed incomplete bisulphite conversion in two samples, which were omitted from downstream analyses. Finally, 2937 probes were removed as they had more than five samples with a bead count of less than three and/or ≥1 % of the samples had a detection *p* value of >0.05. At the end of this filtering process, the dataset comprised 435,889 probes measured in nine ALHs (BD *n* = 5; MDD *n* = 4), ten ULHs and eight MIs (Additional file 1: Table S1).

### Selection of normalisation method

Twelve normalisation methods available in the R package *wateRmelon* [20] were ranked according to their ability to reduce noise attributable to technical error (Additional file 1: Table S2). Daten 2 was identified as the optimum normalisation method.

### Assessment of between-group differences in cell composition

Due to the presence of cell type-specific DNA methylation patterns, individual differences in blood cellular composition can confound the assessment of methylation. As such, between-group differences in estimated cellular proportions were assessed. No significant differences were observed (*p* ≥ 0.276).

### Surrogate variable analysis

To reduce the potentially confounding effects of unmeasured and/or unmodelled variables, surrogate variables (SVs) were estimated [21]. This resulted in the identification of six significant SVs, which were fitted as covariates in the differentially methylated position (DMP) analysis.

### Identification of DMPs

**Fig. 1:**
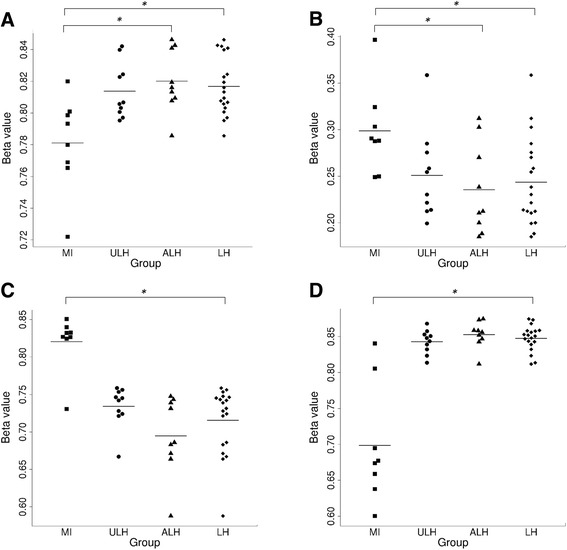
Bee swarm plots showing DNA methylation levels (*β* values) for the four loci identified as being differentially methylated in the LH vs. MI comparison (**a** *FANCI*, **b** *NBEAL2*, **c** *GCOM1/MYZAP*, **d** *AHRR*). Methylation values are shown for the married-in control (*MI*), affected carriers of the linked haplotype (ALH), unaffected carriers of the linked haplotype (ULH) and all linked haplotype carriers (LH) groups. *FDR-adjusted *p* ≤ 0.1

Linear regression analysis was performed to assess DNA methylation at the 435,889 probes retained after data filtering. This analysis revealed four loci to be significantly differentially methylated following multiple testing correction (false discovery rate (FDR)-adjusted *p* ≤ 0.1) when comparing the LH group with the MI group (Table 1; Additional file 1: Table S3; Fig. 1). These loci map to the following: an intron in *FANCI*, 147 bp downstream of *NBEAL2*, the shared promoter region of *GCOM1* and *MYZAP* and an intronic region of *AHRR*. Two of these loci, *FANCI* and *NBEAL2*, were also found to be significantly differentially methylated when comparing the ALH group with the MI group (Table 2; Additional file 1: Table S4; Fig. 1). Comparison of (i) the ULH group with the MI group and (ii) the ALH group with the ULH group did not yield any significant results following multiple testing correction (Additional file 1: Tables S5 and S6).

**Table 1:** Differentially methylated positions identified when comparing the LH group with the MI group

Probe ID	Chr.	Coordinate^a^	Gene	*β* difference^b^	*p* value	Adjusted *p* value^c^
cg12858231	15	89846095	*FANCI*	0.0356	2.54 × 10^−7^	0.0560
cg09354556	3	47051341	*NBEAL2* ^d^	−0.0551	2.99 × 10^−7^	0.0560
cg22708112	15	57883393	*GCOM1/MYZAP*	−0.105	3.85 × 10^−7^	0.0560
cg12251573	5	421644	*AHRR*	0.149	6.58 × 10^−7^	0.0717

^a^hg19/GRCh37

^b^LH β mean-MI β mean

^c^Benjamini-Hochberg false discovery rate-adjusted *p* value

^d^This probe maps 147 bp downstream of *NBEAL2*

**Table 2:** Differentially methylated positions identified when comparing the ALH group with the MI group

Probe ID	Chr.	Coordinate^a^	Gene	*β* difference^b^	*p* value	Adjusted *p* value^c^
cg09354556	3	47051341	*NBEAL2* ^d^	−0.0633	1.93 × 10^−7^	0.0711
cg12858231	15	89846095	*FANCI*	0.0390	3.26 × 10^−7^	0.0711

^a^hg19

^b^ALH β mean-MI β mean

^c^Benjamini-Hochberg false discovery rate-adjusted *p* value

^d^This probe maps 147 bp downstream of *NBEAL2*

### Assessment of FANCI expression

**Fig. 2:**
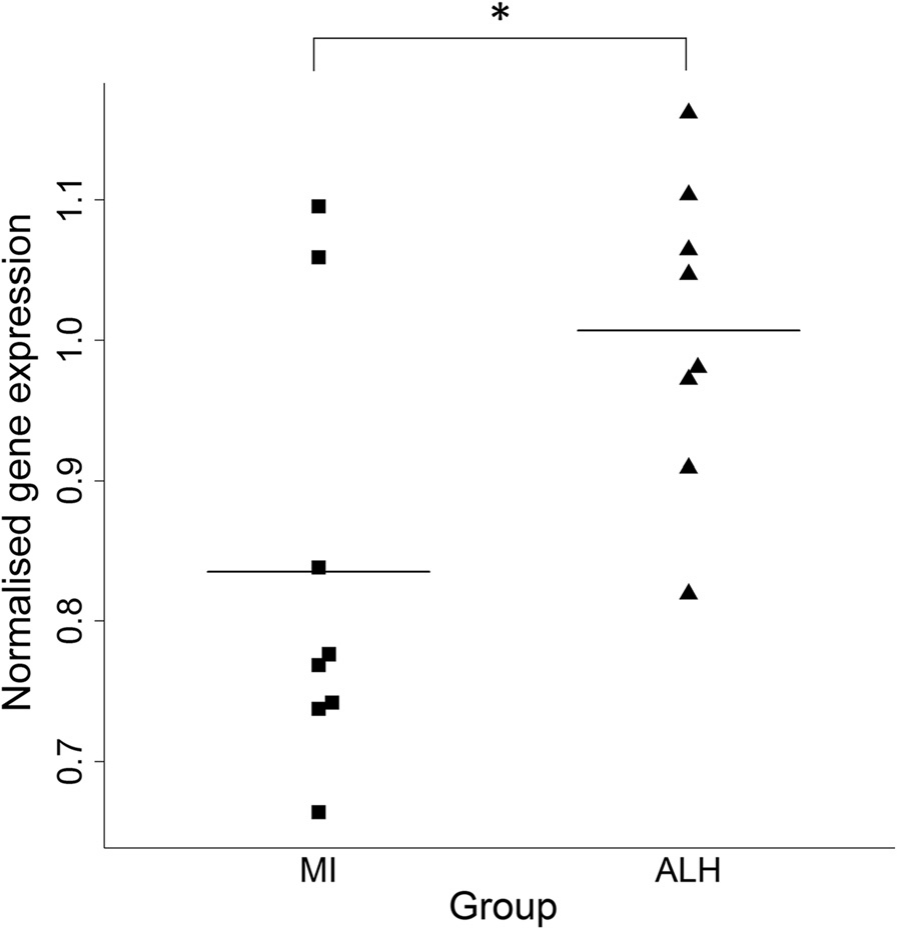
Bee swarm plot showing normalised gene expression values for *FANCI* in the married-in control (*MI*) and affected carriers of the linked haplotype (ALH) groups. *FANCI* expression was measured in lymphoblastoid cell lines using qRT-PCR and was normalised to the geometric mean of *ATP5B*, *RPLP0* and *UBC*. *p ≤ 0.05

As a preliminary step in investigating the consequences of the increase in methylation at a site within *FANCI* observed in the LH and ALH groups (when compared with the MI group), we assessed *FANCI* expression. Expression was measured in lymphoblastoid cell lines (LCLs) obtained from the same individuals who were assessed for DNA methylation. Three individuals (two ALH and one MI) were excluded as they were deemed to be outlier samples. *FANCI* expression was, therefore, compared between eight ALH, ten ULH and eight MIC individuals (Additional file 1: Table S1). A significant increase in *FANCI* expression was observed in the ALH group when compared to the MI group using a linear regression model that covaried for gender (*p* = 0.0423, fold change = 1.21; Fig. 2). A non-significant increase in expression was observed when comparing the LH and MI groups (*p* = 0.206, fold change =1.13).

### Identification of DMRs

Correlation between DNA methylation at neighbouring probes on the 450 K array permits methylation changes to be considered at a regional level [22]. This approach reduces the impact of any individual poor-performing probes, rendering the results more robust. For each comparison (ALH vs. MI, ULH vs. MI, ALH vs. ULH and LH vs. MI), nominally significant probes (*p* ≤ 0.05) from the DMP analyses were assessed for differentially methylated regions (DMRs). Details of the number of probes entered into the analyses and the number of DMRs identified for each comparison are presented in Table 3. The results from these analyses are presented in their entirety in Additional file 1: Tables S7–S10.

**Table 3:** Summary of differentially methylated region (DMR) analyses

Comparison	No. of probes included in DMR analysis^a^	No. of DMRs detected
ALH vs. MI	23,205	150
ULH vs. MI	20,599	120
ALH vs. ULH	16,755	49
LH vs. MI	23,433	156

^a^Nominally significant probes (*p* ≤ 0.05) from the DMP analyses were entered into a modified version of the champ.lasso function implemented in the R package *ChAMP* [73]

DMRs were compared with regions previously implicated in the pathogenesis of BD, MDD or schizophrenia through GWAS. A literature search identified five studies reporting genome-wide significant associations for BD [3, 23–26] and one study reporting genome-wide significant associations for MDD [27]. Genome-wide significant associations for schizophrenia were identified from three studies [28–30]. Four DMRs overlapping with regions previously implicated in schizophrenia were identified [28–30]. These regions map to *CACNB2* (LH vs. MI *p* = 1.84 × 10^−4^), the extended MHC (ULH vs. MI *p* = 1.37 × 10^−4^), *C11orf87* (ALH vs. ULH *p* = 5.32 × 10^−5^) and an intergenic region between *CYP2D7* and *TCF20* (LH vs. MI *p* = 1.49 × 10^−5^; ULH vs. MI *p* = 1.85 × 10^−5^). These regions of overlap are detailed in Table 4.

**Table 4:** Overlap between DMRs and regions implicated in schizophrenia by a recent large-scale GWAS [28]

PGC SCZ region^a^	DMR coordinates^a^	DMR gene(s)	DMR comparison	DMR *p* value	DMR direction^b^
Chr10:18681005-18770105	Chr10:18688985-18689948	*CACNB2*	LH vs. MI	1.84 × 10^−4^	Up
Chr22: 42548710-42548874	Chr22: 42548710-42548874	–	LH vs. MI	1.49 × 10^−5^	Up
			ULH vs. MI	1.85 × 10^−5^	Up
Chr11: 109285471-109610071	Chr11: 109294141-109294239	*C11orf87*	ALH vs. ULH	5.32 × 10^−5^	Up
Chr6: 28303247-28712247	Chr6: 28555640-28559522	Extended MHC	ULH vs. MI	1.37 × 10^−4^	Down

^a^hg19/GRCh37

^b^“Up” indicates hypermethylation and “down” indicates hypomethylation compared to the reference group

Three DMRs are particularly notable for their significance and the involvement of a large number of probes. These are a 829-bp hypomethylated region (spanning 29 probes) located at the developmentally important *HOXA5* locus, observed in the ALH, ULH and LH groups when compared with the MI group (ALH vs. MI *p* = 3.65 × 10^−62^; ULH vs. MI *p* = 5.85 × 10^−61^; LH vs. MI *p* = 1.62 × 10^−77^); a 847-bp hypermethylated region (spanning either 22 (LH) or 24 (ALH) probes) located towards the 5′ end of *RNF39*, observed in the ALH and LH groups when compared with the MI group (ALH vs. MI *p* = 3.45 × 10^−32^; LH vs. MI *p* = 2.11 × 10^−29^) and a 1461-bp hypomethylated region (spanning 22 probes) encompassing the promoter regions and the first exons of *AGPAT1* and *RNF5*, observed in the ALH group compared to the ULH group (*p* = 1.19 × 10^−20^).

### Gene ontology analysis

In order to ascertain whether genes involved in particular biological processes, molecular functions or cellular components were overrepresented amongst the most differentially methylated loci; gene ontology (GO) analysis was performed for each of the four comparisons, ALH vs. MI, ULH vs. MI, ALH vs. ULH and LH vs. MI. This resulted in the identification of several GO categories showing FDR-significant enrichment (*q* ≤ 0.1; Additional file 1: Table S11–S21). Considering those GO categories that are classified as pertaining to “biological processes”, a theme common to all four comparisons was development. Within this broad theme, GO categories relating to the development of the nervous system (e.g. “regulation of nervous system development” (ALH vs. MI *q* = 1.99 × 10^−4^; LH vs. MI *q* = 5.36 × 10^−2^), “neuron projection guidance” (ULH vs. MI *q* = 1.17 × 10^−2^; ALH vs. ULH *q* = 1.40 × 10^−3^) and “nervous system development” (ALH vs. ULH *q* = 3.30 × 10^−4^)) ranked highly for all four comparisons. Continuing the theme of enrichment for neurologically relevant GO categories, both the ALH vs. MI and LH vs. MI comparisons revealed a significant enrichment for genes belonging to the “molecular function” GO category “voltage-gated ion channel activity” (ALH vs. MI *q* = 1.19 × 10^−2^; LH vs. MIC *q* = 6.00 × 10^−2^). Of those GO categories considered to relate to “cellular components”, the LH vs. MI comparison yielded several results relating to neurons and synapses, with the category “neuron part” attaining a *q* value of 7.72 × 10^−2^. Interestingly, in light of the important role believed to be played by calcium signalling in psychiatric illness [31], a significant enrichment for “calcium ion binding” was found when comparing the ALH and ULH groups (*q* = 1.23 × 10^−4^).

## Discussion

Several members of a large Scottish family carry a 20 Mb haplotype on chromosome 4p that confers risk for BD and MDD [17, 18]. Here, we sought to further our understanding of the mechanisms mediating the adverse effects of the LH by studying DNA methylation, an epigenetic mark found to be altered in individuals with psychiatric disorders (e.g. [14, 32–34]). Moreover, as some carriers of the linked haplotype remain well, we aimed to identify changes in DNA methylation that might reflect the involvement of additional risk/protective loci. To the best of our knowledge, this represents the first family-based genome-wide study of DNA methylation in the major affective disorders, BD and MDD. By studying related individuals who share a known genetic susceptibility locus, our study took advantage of the increase in statistical power conferred by the reduced genetic and aetiological heterogeneity expected in a single family compared with larger case-control studies.

Analysis of carriers of the LH resulted in the identification of four significantly differentially methylated loci, when compared with a group of MI. These loci map to an intron in *FANCI*, the *GCOM1* and *MYZAP* promoter region, an intron in *AHRR*, and 147 bp 3′ of *NBEAL2*. The methylation differences at two of these loci, *FANCI* and *NBEAL2*, were also significant when considering only those carriers of the LH who had been diagnosed with either MDD or BD. Although none of these loci showed significant differential methylation when comparing the ALH and ULH groups, it is interesting to note that the mean level of methylation in the ULH group falls between those of the ALH and MI groups for all four loci. This might suggest that possession of the linked haplotype results in a change in methylation that is further modified by additional susceptibility/protective factors.

Increased whole blood *FANCI* methylation was accompanied by increased expression in LCLs from affected carriers of the linked haplotype, further supporting the notion that altered *FANCI* function might contribute to the pathogenic effects of the LH. It is important to note that we were unable to measure methylation and expression in the same tissue. This is a limitation of the present study that precludes us from drawing any definitive conclusions regarding the relationship between altered methylation and expression. Future studies should aim to characterise the relationship between *FANCI* expression and methylation in this family.

*FANCI* is a member of the Fanconi anaemia complementation (*FANC*) gene family. Mutations in these genes can cause Fanconi anaemia, a rare genome instability syndrome. Members of the FANC family act together in the Fanconi anaemia pathway to repair DNA damage. Phosphorylation of FANCI has been shown to act as a trigger for this process [35], and siRNA-induced depletion of FANCI has been shown to induce a higher baseline rate of DNA damage and reduced capacity to mend double strand breaks [36].

Genetic variation in members of the Fanconi anaemia pathway has been implicated in neurodevelopmental phenotypes and psychiatric illness. FANCD2/FANCI-associated nuclease 1 (FAN1) is a repair nuclease that is recruited to sites of interstrand crosslinks by interacting with a FANCD2-FANCI complex. The *FAN1* gene is located at 15q13.3, a region affected by multiple microdeletions that predispose to a number of clinical phenotypes, including schizophrenia [37, 38], autism spectrum disorder (ASD), attention deficit hyperactivity disorder, epilepsy and intellectual disability [39]. An exome sequencing study identified a cluster of rare non-synonymous variants located within a 20-kb window that spans several functional domains of *FAN1*, which were associated with schizophrenia with depressive features, schizoaffective disorder and ASD [40]. More recently, a large-scale GWAS found a genome-wide significant association between the *FANCL* locus and schizophrenia [28].

*GCOM1* forms part of the *GRINL1A* complex transcription unit, which comprises three groups of transcripts [41]. *GCOM1*, which is a read-through transcript of *MYZAP* and *POLR2M*, shows similarity in amino acid sequence to the NR1 n-methyl-d-aspartate (NMDA) subunit-interactor Yotiao [42] and the amino termini of the NR2 and NR3 NMDA subunits [41]. Evidence for an interaction between GCOM1 and NR1 has been identified in the rat brain, where GCOM1 facilitates NMDA receptor activity [43]. The known roles of NMDA receptors in neurodevelopment, neuroplasticity and excitotoxicity [44], together with the evidence implicating altered NMDA function in psychiatric illness [45, 46] render GCOM1 a strong functional candidate. Interestingly, a three nucleotide deletion within *GRINL1A* has been detected in an exome sequencing study of sporadic ASD [47].

We detect decreased methylation at a site that falls within a DNase hypersensitive site (DHS) in the *GCOM1*/*MYZAP* promoter region [48]. DHSs are indicative of an open chromatin structure, which makes DNA accessible to binding by transcription factors. Characterisation of the genomic locations of GWAS-significant variants has revealed that 76.6 % of associated non-coding SNPs are located within DHSs or are in complete linkage disequilibrium with a SNP located in a DHS [5]. This suggests that the observed change in methylation might confer an effect on *GCOM1* and *MYZAP* expression; however, further work is required to assess this possibility and to assess any effects on NMDA receptor activity.

Consideration of our data at a regional level revealed a hypermethylated region located within an intronic region of multiple *CACNB2* isoforms and overlapping the promoter region of a single *CACNB2* isoform in the LH group. Variation at the *CACNB2* locus has been found to increase risk for schizophrenia [28] and for the five psychiatric disorders, schizophrenia, BD, MDD, ASD and attention deficit hyperactivity disorder, included in a recent cross-disorder GWAS [49]. The hypermethylated region overlaps with a DHS and several chromatin immunoprecipitation (ChIP)-identified transcription factor binding sites [48]. Taken together, it is possible that the observed increase in methylation might exert an effect on the expression of the short *CACNB2* isoform; however, this would need to be assessed experimentally.

Comparison of the affected and unaffected carriers of the linked haplotype identified a region of hypomethylation upstream of *HTR2A*. This gene encodes the 5-HT2A receptor, a target of both atypical antipsychotics [50] and selective serotonin reuptake inhibitors [51]. Moreover, variation in *HTR2A* has been associated with risk for MDD by candidate gene studies (reviewed in [52]) and a meta-analysis [53]. Another DMR of interest falls within an intronic region of the mitotic spindle-assembly checkpoint gene *MAD1L1*, adjacent to a region previously associated with risk for schizophrenia and BD [28, 54]. *MAD1L1* has been shown to be a target of another schizophrenia-susceptibility gene, miR-137 [55]. A DMR identified in the comparison of these two groups worth noting for its potential functional relevance affects *NPAS4*. We observed reduced methylation in the ALH group in a region spanning the final exon of *NPAS4. NPAS4* encodes a brain-specific transcription factor, which is involved in regulating the formation of inhibitory synapses [56]. Stress, a well-established risk factor for psychiatric illness [57], has been shown to modulate *NPAS4* methylation in mice [58].

In order to investigate the biological systems potentially impacted by the observed methylation changes, GO analysis was performed. It is interesting to note the presence of enrichment for GO categories pertaining to neurodevelopment and neuronal function in all four comparisons. This finding is particularly pertinent in light of the fact that it was necessary to assess DNA methylation differences relevant to the pathogenesis of BD and MDD in a non-neuronal tissue. Our findings indicate that it may be possible to detect methylation differences relevant to nervous system function in the blood, despite the existence of between-tissue differences in DNA methylation [59, 60].

When performing GO analysis of methylation array data, a single *p* value must be selected to represent each gene. We decided to select the most significant *p* value for any locus within a gene as a methylation change at an individual locus has the potential to confer an effect on gene function. As such, genes that contain more CpG sites are more likely to have their function altered by a change in methylation and are, therefore, more likely to obtain a more significant *p* value. A confounding factor, however, is that genes that are targeted by more probes also have a greater likelihood of obtaining a more significant *p* value by chance due to multiple testing. The conflation of these two factors presents an analytical challenge as correcting for multiple testing risks reducing true biological signal. This is a limitation of currently available methods for GO analysis of methylation data, which must be considered when interpreting our findings.

As each blood cell type has a distinct methylation profile, it was important to assess the existence of systematic between-group variation in cell type distribution, which could confound the detection of methylation differences [61, 62]. We did not observe any between-group differences in estimated cell type proportions, suggesting that variation in cell type distribution would be unlikely to exert a large effect on the differences observed in our sample. Moreover, by performing surrogate variables analysis (SVA), we were able to fit a set of SVs in our analyses that controlled for all unmodelled sources of variation, including cell type distribution [21]. Together, the set of SVs produced by SVA efficiently represent the effects of all unmeasured or unmodelled confounding variables on DNA methylation whilst protecting the primary variable of interest. As such, SVA minimises the number of covariates that must be included, thus helping to avoid model overfitting problems. With regards to modelling the effects of cell type distribution, SVA confers the advantage of negating the need to decide which cell types to include.

Another factor that should be considered when interpreting our findings is that affected carriers of the linked haplotype were ill and taking medication prior to their blood sample being obtained for methylation analysis. As drug treatments for MDD and/or BD have previously been shown to alter DNA methylation [63–67], it is possible that DNA methylation in this group was affected by medication. To the best of our knowledge, however, methylation at the sites found to be differentially expressed in the current study has not been shown to be affected by relevant drug treatments [63, 66, 67]. Moreover, the fact that the mean methylation level in the ULH group falls between the means of the MI and ALH groups for the four significant LH vs. MI loci argues against medication being a primary driver of our results.

The small size of the sample studied here is likely to represent a limitation of the study. Although our approach of studying a large family benefits from reduced aetiological heterogeneity, it is likely that some changes remain undetectable due to insufficient power. In an attempt to maximise our chances of detecting illness-relevant changes in methylation, we used a significance threshold of FDR-adjusted *p* ≤ 0.1. It is, of course, possible, however, that some of our results are false positives. Future studies should expand upon the work presented here, perhaps by leveraging the increased power conferred by the study of a relevant quantitative phenotype. Ultimately, the synthesis of results obtained through family studies with the results of larger case-control studies, together with downstream functional analyses are likely to be important steps in unravelling the epigenetic contribution to psychiatric disorders.

## Conclusions

We have found evidence for altered blood DNA methylation in carriers of a haplotype linked to BD and MDD. Many affected genes have neuronal roles, participating in neurodevelopment and ion channel activity. Our observation that significant single locus methylation differences are found in haplotype carriers regardless of affectation status is consistent with our previous finding of reduced cognitive function in haplotype carriers but no difference in cognitive function between affected and unaffected carriers of the haplotype [68]. Nevertheless, through gene ontology analysis, we did find evidence that differences in DNA methylation between affected and unaffected haplotype carriers converge on neurologically relevant functions. Taken together, our findings suggest a scenario whereby the presence of the linked haplotype confers multiple effects on DNA methylation, which are in turn modified by additional genetic and/or environmental influences that fine-tune an individual’s risk of developing a mood disorder.

## Methods

This study was approved by the Multicentre Research Ethics Committee for Scotland, and appropriate informed consent was obtained from the human subjects.

### Sample

The individuals included in this study were selected from a previously described large Scottish family multiply affected by BD or MDD [17, 18]. A ~20 Mb haplotype located on chromosome 4p has previously been found to segregate with illness in this family with a maximum LOD score of 4.41 [18]. Carriers of the haplotype were grouped according to illness-status, resulting in two groups: affected (either BD or MDD) carrier of the disease-linked haplotype (ALH) and unaffected carrier of the disease-linked haplotype (ULH). An additional group of well married-in controls (MI) who do not carry the disease-linked haplotype was included for comparison. In total, ten ALH, ten ULH and nine MI individuals were assessed.

### Extraction of blood DNA

Blood (9 ml) was collected in an EDTA tube. DNA was extracted at the Wellcome Trust Clinical Research Facility at the University of Edinburgh, using the Nucleon BACC2 Genomic DNA Extraction Kit (Thermo Fisher Scientific, Loughborough, UK), following the manufacturer’s instructions.

### Genome-wide methylation profiling

Whole blood genomic DNA (500 ng) was treated with sodium bisulphite using the EZ-96 DNA Methylation Kit (Zymo Research, Irvine, California), following the manufacturer’s instructions. DNA methylation was assessed using the Infinium HumanMethylation450 BeadChip (Illumina Inc, San Diego, California), according to the manufacturer’s protocol. Samples were assigned to chips such that, as far as possible, group (ALH, ULH or MI) and gender were counter-balanced across chips.

The resultant raw intensity (.idat) files were read into R using the *minfi* package [69], which was used to perform initial quality control assessments. Subsequently, filtering of poor-performing samples and sites was performed. Samples were removed from the dataset if: (i) they failed any of the quality control assessments carried out in *minfi* or (ii) ≥1 % sites had a detection *p* value of >0.05. Probes were removed from the dataset if: (i) they were located within two base pairs of SNP with a minor allele frequency of ≥5 %; (ii) they were predicted to cross-hybridise [19] and (iii) they had more than five samples with a bead count of less than three or (iv) ≥1 % samples had a detection *p* value of >0.05.

The data was normalised using the Daten 2 method, selected using the R package *wateRmelon* [20]. Daten 2 involves adjusting the background difference between type I and type II assays (by adding the offset between type I and II probe intensities to type I intensities). A linear model is incorporated at this stage to eliminate positional effects. Between-array quantile normalisation is then performed for the methylated and unmethylated signal intensities separately (type I and type II assays normalised together).

Prior to downstream analyses, M-values, defined as M-value = log2((*M* + 100)/(*U* + 100)), where *M* represents the methylated signal intensity and *U* represents the unmethylated signal intensity, were calculated for the normalised data. For ease of interpretation, the data were converted to β-values (β-value = 2^*M*^/(2^*M*^ + 1)) prior to presentation.

### Assessment of between-group differences in the whole blood cellular composition

In order to assess between-group differences in the cellular composition of whole blood samples, estimated cell counts for B-lymphocytes, granulocytes, monocytes, natural killer cells, CD4+ T-lymphocytes and CD8+ T-lymphocytes were generated using the estimate CellCounts function in *minfi*. This function implements Jaffe and Irizarry’s [62] modified version of Houseman’s [61] algorithm. Between-group differences in cell composition were assessed using Student’s *t* tests. A *p* value of ≤0.05 was deemed to represent a significant between-group difference.

### Identification of significant surrogate variables

DNA methylation can be affected by many sources of variation, and it is, therefore, important to account for these variables when assessing differential methylation. A complicating factor is that many sources of variation are unknown or unmeasured. Moreover, even when potential sources of variation are measured, it can be unclear how best to model these potential confounding variables [21]. Surrogate variable analysis (SVA) identifies a set of significant SVs that together represent variation in DNA methylation that is not attributable to the primary variable of interest. These SVs are then fitted as covariates in the linear models implemented to identify DMPs. Covarying for the identified SVs controls for sources of unmeasured/unmodelled variation (e.g. age, genetic relatedness, smoking status, cell composition), which might otherwise confound the relationship between DNA methylation and the independent variable of interest [21, 70]. A key advantage of SVA is that it permits complex relationships between confounders and DNA methylation, for example, interactions between multiple confounding variables. SVA was carried out using the “be” method with the R package SVA [71].

### Identification of differentially methylated positions

DMPs were then identified using the R package *limma* [72] by fitting linear models with the outcome variable “M-value” and the predictor variables “group” (ALH, ULH or MI) and “gender”, together with the significant SVs identified by SVA. The following comparisons were carried out as follows: (i) ALH and MI, (ii) ULH and MI, (iii) ALH and ULH and (iv) a combined linked haplotype carrier group (ALH and ULH; henceforth referred to as LH) and MI. Correction for multiple testing was implemented using the Benjamini-Hochberg false discovery rate (FDR), with adjusted *p* values of ≤0.1 deemed to be significant.

### Identification of differentially methylated regions

Differentially methylated regions (DMRs) were identified using a modified version of the champ.lasso function implemented in the R package *ChAMP* [73]. DMRs were defined as regions containing three or more adjacent probes within a defined lasso region showing unidirectional changes in methylation that attained nominal significance (unadjusted *p* ≤ 0.05) in the DMP analysis. The lasso region was set to 2 kb and was scaled according to the local genomic/epigenomic landscape in order to account for uneven probe spacing across the genome [73]. DMRs were merged with neighbouring DMRs where they were separated by less than 1 kb, using the “minDmrSep” parameter in the champ.lasso function. *P* values were estimated for each DMR as described by Butcher et al. [73]. Briefly, Stouffer’s method was used to combine individual probe *p* values, which were weighted by the underlying correlation structure of the M- values. *P* values from correlated probes were down-weighted whilst *p* values from uncorrelated probes were up-weighted. DMRs with *p* values meeting a Benjamini-Hochberg FDR-corrected threshold of ≤0.05 were included in the final DMR lists.

### Comparison of DMRs with GWAS results

DMRs were assessed for overlap with regions previously implicated in BD, MDD and the related condition, schizophrenia, by GWAS. A literature search was carried out using PubMed (on 7 October 2015) to identify case-control GWAS studies and GWAS meta-analyses involving individuals with either BD, MDD or schizophrenia. Only GWAS results attaining genome-wide significance (*p* ≤ 5 × 10^−8^) were considered. Significantly associated genes/regions from the GWAS studies were defined as per the original study. Overlap was defined as either complete or partial overlap between the DMR and a GWAS associated gene/region.

### Lymphoblastoid cell line culture and extraction of RNA

Human Epstein-Barr virus (EBV)-transformed LCLs were derived from blood lymphocytes obtained from family members at the European Collection of Cell Cultures (http://www.phe-culturecollections.org.uk/collections/ecacc.aspx). LCLs were maintained in Roswell Park Memorial Institute medium with 10 % foetal bovine serum at 37 °C and 5 % CO_2_.

Total RNA was extracted using the RNeasy Mini Kit (Qiagen, Manchester, UK), according to the manufacturer’s instructions. An on-column DNase digest step was performed using the RNase-Free DNase Set (Qiagen, Manchester, UK), according to the manufacturer’s instructions. RNA quantity and quality were assessed using the Agilent Bioanalyzer at the WTCRF. The Agilent Bioanalyzer reports RNA integrity numbers (RINs), which indicate how intact an RNA sample is (1 = completely degraded, 10 = completely intact). Sample RINs ranged from 8.6 to 10, indicating that the RNA was suitable for use in qRT-PCR [74].

### qRT-PCR assessment of Fanconi anaemia, complementation group I (FANCI) expression

RNA (1 μg) was reverse transcribed using the Transcriptor First Strand cDNA Synthesis Kit (Roche) using random hexamer primers, according to the manufacturer’s instructions.

cDNA samples were diluted by a factor of 1/100. *FANCI* expression was assessed using a TaqMan Gene Expression Assay (Hs01105308_m1, Applied Biosystems by Thermo Fisher, Loughborough, UK). Briefly, 4.5 μl cDNA was added to 5 μl TaqMan Universal PCR Master Mix, No AmpErase UNG (2×) (Applied Biosystems) and 0.5 μl 20× TaqMan Gene Expression Assay (Applied Biosystems by Thermo Fisher, Loughborough, UK) in a 384-well plate. qPCRs were performed on a 7900HT PCR system with the following assay conditions: 15 s at 95 °C, followed by 1 min at 60 °C for 40 cycles. *FANCI* expression was normalised to the geometric mean of the expression levels of three reference genes, *ATP5B*, *RPLP0* and *UBC*, which were selected from an initial set of seven using geNorm [75]. Assay details are described in Additional file 1: Table S22. Each sample was measured in technical triplicates and the mean of this triplicate used in downstream analyses. Outlier samples, defined as data points falling outside of the range defined by median ± 1.5 × inter-quartile range were excluded.

Differences in *FANCI* expression were assessed by linear regression, covarying for gender. A *p* value of ≤0.05 was deemed to be significant.

### Gene ontology analysis

Gene symbols representing all genes targeted by reliably detected probes (*n* = 17,686 genes) were ranked according to the probe *p* value calculated when identifying DMPs (where an individual gene was targeted by multiple probes, the best *p* value was retained for GO analysis) and ranked-list GO analysis performed using GOrilla [76, 77]. GOrilla performs a hypergeometric test to assign a *p* value to each GO category and then calculates the Benjamini-Hochberg FDR to reflect the number of GO categories assessed. GO categories with a *q* value ≤0.1 were considered statistically significant.

## Additional file

Additional file 1:
**Table S1. **Sample demographic information for the individuals included in this study. **Table S2**. Comparison of 12 normalisation methods. **Table S3**. Probes attaining an uncorrected *p*-value of ≤ 0.05 in the comparison of individuals carrying the linked haplotype (LH) and married in controls (MI), ranked by *p*-value. **Table S4**. Probes attaining an uncorrected *p*-value of ≤ 0.05 in the comparison of affected individuals carrying the linked haplotype (ALH) and married in controls (MI), ranked by *p*-value. **Table S5**. Probes attaining an uncorrected *p*-value of ≤ 0.05 in the comparison of unaffected individuals carrying the linked haplotype (ULH) and married in controls (MI), ranked by *p*-value. **Table S6**. Probes attaining an uncorrected *p*-value of ≤ 0.05 in the comparison of affected individuals carrying the linked haplotype (ALH) and unaffected carriers of the linked haplotype (ULH), ranked by *p*-value. **Table S7**. Differentially methylated regions (DMRs) identified in affected carriers of the linked haplotye(ALH) compared to married in controls (MI), ranked by *p*-value. **Table S8**. Differentially methylated regions (DMRs) identified in carriers of the linked haplotye (LH) compared to married in controls (MI), ranked by *p*-value. **Table S9**. Differentially methylated regions (DMRs) identified in unaffected carriers of the linked haplotye (ULH) compared to married in controls (MI), ranked by *p*-value. **Table S10**. Differentially methylated regions (DMRs) identified in affected carriers of the linked haplotye (ALH) compared to unaffected carriers of the linked haplotype (ULH), ranked by *p*-value. **Table S11**. Significantly enriched molecular process gene ontology (GO) categories identified in the ALH vs. MI comparison. **Table S12**. Significantly enriched biological function gene ontology (GO) categories identified in the ALH vs. MI comparison. **Table S13**. Significantly enriched cellular component gene ontology (GO) categories identified in the ALH vs. MI comparison. **Table S14**. Significantly enriched molecular process gene ontology (GO) categories identified in the ULH vs. MI comparison. **Table S15**. Significantly enriched biological function gene ontology (GO) categories identified in the ULH vs. MI comparison. **Table S16**. Significantly enriched cellular component gene ontology (GO) categories identified in the ULH vs. MI comparison. **Table S17**. Significantly enriched molecular process gene ontology (GO) categories identified in the ALH vs. ULH comparison. **Table S18**. Significantly enriched biological function gene ontology (GO) categories identified in the ALH vs. ULH comparison. **Table S19**. Significantly enriched molecular process gene ontology (GO) categories identified in the LH vs. MI comparison. **Table S20**. Significantly enriched biological function gene ontology (GO) categories identified in the LH vs. MI comparison. **Table S21**. Significantly enriched cellular component gene ontology (GO) categories identified in the LH vs. MI comparison. **Table S22**. Details of the qRT-PCR assays used to measure the seven reference genes assessed for the stability of their expression using geNorm [75]. XLSX 8646 kb)

## Abbreviations

ALH: affected carrier of the linked haplotype
ASD: autism spectrum disorder
BD: bipolar disorder
DHS: DNase hypersensitive site
DMP: differentially methylated position
DMR: differentially methylated region
FDR: false discovery rate
GO: gene ontology
GWAS: genome-wide association study
LCL: lymphoblastoid cell line
LH: all carriers of the linked haplotype
MDD: major depressive disorder
MI: married in control
NMDA: n-methyl-d-aspartate
qPCR: quantitative polymerase chain reaction
RIN: RNA integrity number
SV: surrogate variable
SVA: surrogate variables analysis
ULH: unaffected carrier of the linked haplotype
